# New *JAK3-INSL3* Fusion Transcript—An Oncogenic Event in Cutaneous T-Cell Lymphoma

**DOI:** 10.3390/cells12192381

**Published:** 2023-09-29

**Authors:** Loka Reddy Velatooru, Cheng Hui Hu, Pedram Bijani, Xiaohong Wang, Pierr Bojaxhi, Hao Chen, Madeleine Duvic, Xiao Ni

**Affiliations:** Department of Dermatology, The University of Texas MD Anderson Cancer Center, Houston, TX 77030, USA; lvelatooru@mdanderson.org (L.R.V.); chenghui.hu@pfizer.com (C.H.H.); b.pedram@yahoo.com (P.B.); xwang45@mdanderson.org (X.W.); pbojaxhi@gmail.com (P.B.); ch76ch@163.com (H.C.); mduvic@mdanderson.org (M.D.)

**Keywords:** cutaneous T-cell lymphomas, JAK3, INSL3, RNA interference, mycosis fungoides, Sézary syndrome

## Abstract

Constitutively activated tyrosine kinase JAK3 is implicated in the pathogenesis of cutaneous T-cell lymphomas (CTCL). The mechanisms of constitutive JAK3 activation are unknown although a *JAK3* mutation was reported in a small portion of CTCL patients. In this study, we assessed the oncogenic roles of a newly identified *JAK3-INSL3* fusion transcript in CTCL. Total RNA from malignant T-cells in 33 patients with Sézary syndrome (SS), a leukemic form of CTCL, was examined for the new *JAK3-INSL3* fusion transcript by RT-PCR followed by Sanger sequencing. The expression levels were assessed by qPCR and correlated with patient survivals. Knockdown and/or knockout assays were conducted in two CTCL cell lines (MJ cells and HH cells) by RNA interference and/or CRISPR/Cas9 gene editing. SS patients expressed heterogeneous levels of a new *JAK3-INSL3* fusion transcript. Patients with high-level expression of *JAK3-INSL3* showed poorer 5-year survival (n = 19, 42.1%) than patients with low-level expression (n = 14, 78.6%). CTCL cells transduced with specific shRNAs or sgRNAs had decreased new *JAK3-INSL3* fusion transcript expression, reduced cell proliferation, and decreased colony formation. In NSG xenograft mice, smaller tumor sizes were observed in MJ cells transduced with specific shRNAs than cells transduced with controls. Our results suggest that the newly identified *JAK3-INSL3* fusion transcript confers an oncogenic event in CTCL.

## 1. Introduction

Cutaneous T-cell lymphomas (CTCL) are a heterogeneous group of extranodal non-Hodgkin’s lymphomas that first present as cutaneous lesions. Mycosis fungoides (MF) and Sézary syndrome (SS) are the most common forms of CTCL accounting for 65% [1] (MF/SS CTCL). MF usually first manifests as skin patches, plaques, and tumors but it may progress to involve the lymph nodes and blood (some evolving to SS). SS is defined by erythroderma, pruritus, adenopathy, and the presence of more than 1000 cells/μL of Sézary cells in the blood [2]. Malignant T-cells in MF lesions and Sézary cells in the blood are mostly immunophenotypically CD3^+^CD4^+^CD26^−^ and/or CD3^+^CD4^+^CD7^−^ T-cells [3,4]. The genomic etiology of CTCL has not been fully understood which limits our ability to prevent, detect, and treat CTCL. With the development of next generation sequencing over the past years, some new gene mutations in CTCL have been identified [5,6,7,8,9]. However, incidences of these gene mutations are relatively low and none of them have been demonstrated to be dominant oncogenic drivers. Fusion genes have been recognized as important drivers of cancer and are a mechanism for oncogene activation in leukemia and lymphoma [10,11]. Oncogenic fusion genes can be caused by a translocation between chromosomes and can also occur through trans-slicing or read-through events. Fusion genes frequently affect tyrosine kinases or transcription factors, and can cause their constitutive activation, enhancement of downstream signaling, and tumor development [12,13,14]. Our recent transcriptome analysis by RNAseq identified multiple in-frame fusion genes (*C15orf57-CBX3*, *MDD22-SURF6*, *CD28-CTLA4*, etc.) in sorted malignant T-cells from SS patients [5]. Although most of fusion genes are rare events, *JAK3-INSL3* fusion transcripts were observed in more than one third of our patients.

The *JAK3* gene is located on human chromosome *19p13.1* and comprises 24 exons [15]. Three splice isoforms or variants, JAK3S, JAK3B, and JAK3M, have been previously reported [16]. The commonly described isoform, JAK3S or JAK3, has 23 exons and encodes an 1124-amino acid protein. JAK3 is predominantly expressed in hematopoietic tissues and plays an important role during normal lymphocyte development [15,16]. JAK3 is an intracellular non-receptor tyrosine kinase and mediates the signal initiated by cytokines which use the common gamma chain (γc), including IL-2, IL-4, IL-7, IL-9, IL-13, IL-15, and IL-21 [15,17]. Studies have found that many of these cytokines were abnormal and implicated in the pathogenesis of CTCL [18,19,20,21]. The binding of JAK3 to the common gamma chain of cytokine receptors is an upstream of signal transducers and activators of transcription (STAT) 5 and 6 pathway, which activates NFκB leading to lymphopoiesis, differentiation, and more [22,23]. 

The constitutive JAK3 activation has been found in CTCL and implicated its pathogenesis [15,24]. A study reported that a JAK3A572V mutation was found in 1 of 30 CTCL patients, which may be related to constitutive JAK3 activation [25]. However, the mechanisms underlying the constitutive JAK3 activation in CTCL is largely unknown. This study was to test the hypothesis that a newly identified *JAK3-INSL3* fusion transcript may contribute to constitutive JAK3 activation and lymphomagenesis in MF/SS CTCL. To test our hypothesis, we not only assessed the correlation between the expression of the newly identified *JAK3-INSL3* fusion transcript with clinical data from SS patients, but also conducted in vitro and in vivo experiments with established CTCL cell lines. Our findings from patients and cell lines support our hypothesis and the newly identified *JAK3-INSL3* fusion may be an oncogenic event in MF/SS CTCL.

## 2. Materials and Methods

### 2.1. Patients

This study was conducted according to the guidelines of the Declaration of Helsinki, and the protocol was approved by the Institutional Review Board of the University of Texas MD Anderson Cancer Center (Protocol-PA14-0177 was approved on 4 March 2014. All patients in this study are part of our previous reported genomic study [5]. The demographics of 33 patients were provided in Table S1. 

### 2.2. Cell Lines

Human CTCL cell lines, MJ, HuT 78, and HH cells, were purchased from the American Type Culture Collection (Rockville, MD, USA). MyLa, SeAx, H9, PB2S, SZ4, Hut102, and Mac2A cells were kindly provided by Dr. Ivan Litvinov (Department of Medicine, McGill University, Montreal, QC, Canada). Cells were cultured as previously reported [26,27].

### 2.3. Reverse Transcription-Polymerase Chain Reaction (RT-PCR) and Sanger Sequencing

The total RNA of CD4^+^ T cells from SS patients (n = 33) and healthy donors (n = 7) were remaining samples from the previous study [5]. After DNase I treatment, the first-strand cDNA was synthesized from total RNA (200 ng/sample) with an oligo (dT) 12–18 primer using Superscript IV reverse transcriptase (Life Technologies Inc., Gaithersburg, MD, USA). With primers spanning the break points (Table S3), PCR was then performed, and targeted PCR bands were purified for Sanger sequencing. Sequence data were viewed/interpreted using Finch TV chromatogram viewer (Geospiza, Inc. Seattle, WA, USA).

### 2.4. Long-Range PCR

Long-range PCR was performed with primers in Exon 21 of JAK3 and Exon 1 of INSL3 to amplify 8000~10,000 base pair fragments (Table S3) using genomic DNA from SS patients, healthy donors, and CTCL cell lines (MJ, HuT 78, and HH). PCR products were then electrophoresed on 0.8% agarose gel, and the gel images were recorded by a LI-COR Odyssey imager (LI-COR Biosciences, Lincoln, NE, USA).

### 2.5. Quantitative Real-Time PCR or qPCR 

As outlined above in regular RT-PCR, the total RNA from patients, healthy donors, and CTCL cell lines were treated with DNase I and followed by the first-strand cDNA synthesis. Pre-formulated TaqMan primers and probes for INSL3 (Hs01895076_s1) and JAK3 (Hs00169663_m1), and customized primers and probes for *JAK3-INSL3* (AIMSIVI) were used for quantitative real-time PCR. GAPDH (Hs99999905_m1) was used as endogenous control gene. The ABI Prism 7000 Sequence Detection System was used with the default protocol by the manufacturer (Applied Biosystems, Foster City, CA, USA). The relative levels of mRNA expression were quantitated based on the Ct value and then normalized to GAPDH. Relative fold changes were finally calculated [26]. 

### 2.6. ViewRNA^TM^ ISH Cell Assay

To detect *JAK3-INSL3* fusion transcript targets in situ in cells, we employed a direct fluorescence RNA in situ hybridization method—ViewRNA™ ISH Cell Assay (ThermoFisher Scientific, Waltham, MA, USA). This method enabled us to simultaneously detect both JAK3 and INSL3 RNA targets at single copy sensitivity and single cell resolution using a fluorescence microscope. In brief, after cells were fixed and permeabilized, the JAK3-specific probe (type 1 dye, fluorescence label excitation at 550 nm, red) and the INSL3-specific probe (type 4 dye, fluorescence label excitation at 480 nm, green) were added to hybridize to targets. DAPI was added to stain nuclei in blue. The *JAK3-INSL3* fusion transcripts in situ in MJ cells were visualized and pictured using fluorescence microscopes (Olympus FV1000, Olympus Life Science, Waltham, MA, USA) of our Flow Cytometry and Cellular Imaging Core Facility. 

### 2.7. Lentivirus Transduction

Long-term MISSION^®^ *INSL3* shRNA lentiviral transduction particles (Sigma Aldrich, St. Louis, MO, USA) were using for gene silencing. The sequences of shRNAs (TRCN0000118958, TRCN0000118959, TRCN0000118960, TRCN0000118961, and TRCN0000438118) were provided in Table S4. MISSION^®^ pLKO.1-puro control transduction particles (SHC001V) and MISSION^®^ shRNA non-target control transduction particles (SHC002V) were included as negative controls. Briefly, MJ and HH cells were seeded on Day 1 with a complete medium and transduced with lentivirus at a 10 or 5 multiplicity of infection (MOI) on Day 2. After 18–24 h, the virus-containing medium was removed and replaced with a complete medium for 72 h. On Day 5, the cells were incubated with 3 μg/mL puromycin for 48–72 h to select transduced cells. On Days 7–8, the cells were collected, and the knockdown efficiency was assessed using qPCR and/or Western blot analysis. For longer stable transduction, puromycin-containing medium was replenished every 3–4 days (for an additional 1–2 weeks), and the cells were then harvested and assayed.

### 2.8. Cell Viability

CellTiter-Glo^®^ Luminescent Cell Viability Assay was used to measure the cell viability for transduced MJ and HH cells according to the manufacturer’s instructions (Promega, Madison, WI, USA). Briefly, MJ and HH cells transduced with *INSL3* shRNAs or non-target shRNA lentiviral transduction particles were seeded at a density of 2000 cells per well in duplicate in white opaque 96-well microplates in 50 μL of medium. After incubation at 37 °C for 3 h, an equal volume of CellTiter-Glo^®^ reagent was added to each well. The cells were cultured for 24 h and/or 48 h, and the luminescence was measured by BioTek Synergy 2 plate reader (Agilent Technologies, Inc. Santa Clara, CA, USA). 

### 2.9. Soft Agar Colony Formation Assay

A soft agar colony formation assay was used to assess the anchorage-independent growth ability of our transduced MJ and HH cells according to the literature with some modifications [28]. In brief, a bottom layer of 0.7% soft agar with complete media was poured and solidified first (in a 6-well plate or 12-well plate), followed by an upper layer of 0.35% soft agar containing different transduced MJ and HH cells (15,000 cells/well in a 6-well plate or 6000 cells/well in a 12-well plate) suspended in a medium–agar mixture with triplicates. The medium was refreshed every three days for two weeks. After two weeks of incubation, the colonies were stained with 0.01% (*v*/*v*) of crystal violet. The numbers of stained colonies were counted and pictured using a bright field microscope. Besides the numbers of total colonies, the small, medium, and large colonies were also counted, respectively.

### 2.10. Western Blotting Analysis

Equal amounts of cellular proteins (10 or 15 μg) prepared from MJ and HH cells transduced with *INSL3* shRNAs or non-target shRNA lentiviral transduction particles were separated by 4–12% SDS-PAGE gel and electro-transferred onto nitrocellulose membranes. The membranes were blocked with 5% BSA in TBST (Tris-buffered saline, 0.1% Tween 20) for 1 h at room temperature, then incubated overnight with primary antibodies at 4 °C overnight. The primary antibodies and dilutions used were listed in Table S6. After washing with TBST, the membranes were incubated with horseradish peroxidase-conjugated secondary antibodies for 1 h at room temperature. Protein bands were visualized using the SuperSignal West Pico Chemiluminescence Substrate kit (Thermo, Rockford, IL, USA). The equivalent loading of proteins in each well was confirmed by using pan-actin [26].

### 2.11. Proteome Profiler Human Cytokine Array

A Proteome Profiler Human Cytokine Array Kit (R&D Systems, Minneapolis, MN, USA) was used to analyze 36 cytokines/chemokines according to the manufacturer’s protocol. Briefly, lysates were prepared from MJ cells transduced with *INSL3* shRNA -TRC58, -TRC59, -TRC61 or non-target shRNA SHC002V. Lysates (280 μg per sample) were mixed with a cocktail of biotinylated detection antibodies and then incubated with the array membrane which is spotted in duplicate with capture antibodies to specific target proteins. Captured proteins were visualized using chemiluminescent detection reagents. The positive signals seen on developed film were identified by placing the transparency overlay on the array image and aligning it with the three pairs of positive control spots in the corners of each array. The densities of positive cytokines detected were quantified using ImageJ software (NIH). The levels of cytokines/chemokines were compared between *INSL3* shRNA-transduced cells and control-transduced cells, which were considered as 100%.

### 2.12. Xenograft Tumor Formation and Growth in NSG Mice 

All animal experiments in this study were approved by the Animal Care and Use Committee of the University of Texas MD Anderson Cancer Center (ACUF00000375). Female 6–7-week-old NSG mice (NOD.Cg-Prkdc scid Il2rg tm1Wjl/SzJ) were obtained from the Jackson Laboratory (Bar Harbor, ME). In brief, MJ cells transduced with *INSL3* shRNA-TRC58, -TRC61, or non-target shRNA-SHC002V were subcutaneously injected into NSG mice (3.8 × 10^6^/site, 3 mice/group). Tumor formation was monitored twice a week or as needed until the first tumor was observed on Day 20. Tumor growth was monitored and measured twice a week, and tumor volumes were calculated using the formulas—1/2 (length × width^2^) based on manual measurements. On Day 40, the mice were sacrificed, and the tumors were collected and weighted. The tumors were processed and embedded in paraffin blocks. The sections were cut and stained with hematoxylin and eosin (H&E) for histological evaluation. Ki67 protein expression was analyzed using immunohistochemistry.

### 2.13. Statistical Analysis 

Statistical analyses were mostly performed using GraphPad Prism9 software (GraphPad Software, La Jolla, CA, USA). Data were expressed as means ± standard deviations (SD), and statistical analyses were carried out using a *t*-test, Chi-squared test, or analyses of variance (ANOVAs) as needed. The *p*-value ≤ 0.05 was considered statistically significant, and the *p*-value ≤ 0.01 was considered statistically very significant. The survival analysis was conducted to correlate the expression level of new *JAK3-INSL3* fusion transcript with the patient’s overall survival. A Gehan-Breslow-Wilcoxon test was used for the significance.

## 3. Results

### 3.1. The High Prevalence of JAK3-INSL3 Fusion Transcript in SS Patients 

Our recent transcriptome analysis using RNA-seq detected not only tens of in-frame intra- and inter-chromosomal fusion transcripts in malignant T-cells from SS patients, but also multiple fusion transcripts from alternative splicing [5]. Although most of these fusion transcripts were rare, two of *JAK3-INSL3* fusion transcripts were found in multiple patients (Table S2). Due to the crucial roles of JAK3 in hematopoietic cells and hematological diseases, we further verified *JAK3-INSL3* fusion transcripts. 

**Figure 1:**
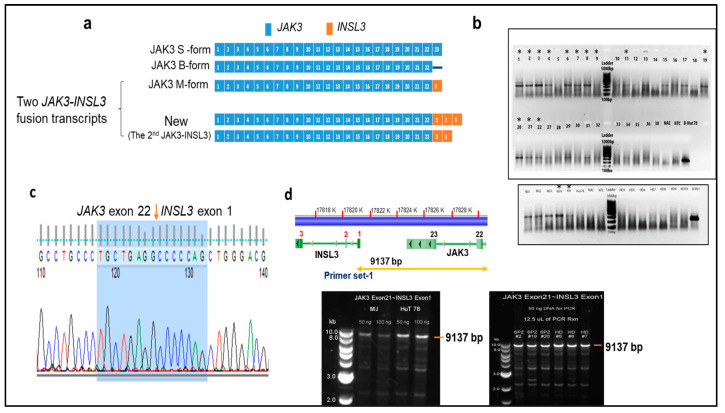
Identification of new *JAK3-INSL3* fusion transcript in SS patients. (**a**) Schematic representation of three JAK3 splice isoforms and two *JAK3-INSL3* fusion transcripts. The first *JAK3-INSL3* fusion transcript (same as JAK3M)—INSL3 exon 3 is fused to JAK3 exon 22; new or the second *JAK3-INSL3* fusion transcript—INSL3 exon 1 is fused to JAK3 exon 22. (**b**) The new *JAK3-INSL3* fusion transcript was amplified by RT-PCR using total RNA of 33 SS patients, 7 healthy donors (HD), and 3 CTCL cell lines (MJ, HH, and HuT 78); (**c**) PCR products were purified for Sanger sequencing, and fusion sequences were confirmed in 13 SS patients and 2 cell lines (indicated in (**b**) with *); (**d**) Long-range PCR was performed to amplify 9137 bp fragment between JAK3 exon 21 and INSL3 exon 1 using genomic DNA from the CD4^+^ T-cells of SS patients, healthy donors, and MJ and HuT of which there were 78 cell lines.

There are three splice isoforms or variants of JAK3 (JAK3S, JAK3B, and JAK3M) that have been previously reported (Figure 1a) [16]. JAK3S or JAK3, the commonly described isoform or variance expressed in hematopoietic cells, is composed of 23 exons and encodes an 1124-amino acid protein. JAK3M is a splice isoform or variant composed of exon 1- 22 of *JAK3* and exon 3 of *INSL3*. JAK3M was previously reported in a group of cell lines [16], and recently detected in T-cell lymphoblastic lymphoma [11]. One of two *JAK3-INSL3* fusion transcripts that we detected had the identical composition to JAK3M. However, the 2nd *JAK3-INSL3* fusion transcript we detected is new: Exon 1 of *INSL3* is fused to exon 22 of *JAK3* as shown in Figure 1a. This second *JAK3-INSL3* fusion transcript was rarely reported and not reported in CTCL. Using total RNA from malignant T-cells of all 33 patients, we ran RT-PCR with primers spanning the fusion site (Table S3, Figure 1b). The PCR products were then Sanger sequenced, and the second *JAK3-INSL3* fusion transcript was confirmed in 13 of 33 SS patients (39.4%) (Figure 1c). 

To clarify whether this newly identified *JAK3-INSL3* fusion transcript results from genomic DNA rearrangements or from alternative splicing, we performed a long-range PCR with a set of primers spanning from JAK3 exon 21 to INSL3 exon1 (Figure 1d, Table S3) using genomic DNA from malignant T-cells. As shown in Figure 1d, we were able to amplify 9137 bp fragments, as expected in HD, in patients (as well as in CTCL cell lines). These results suggest that the second *JAK3-INSL3* fusion transcript detected in malignant T-cells of CTCL patients are from alternative splicing, not from genomic DNA rearrangement. 

### 3.2. The Correlation of the New JAK3-INSL3 Fusion Transcript with Patient’s Clinical Manifestations and Survival 

**Figure 2:**
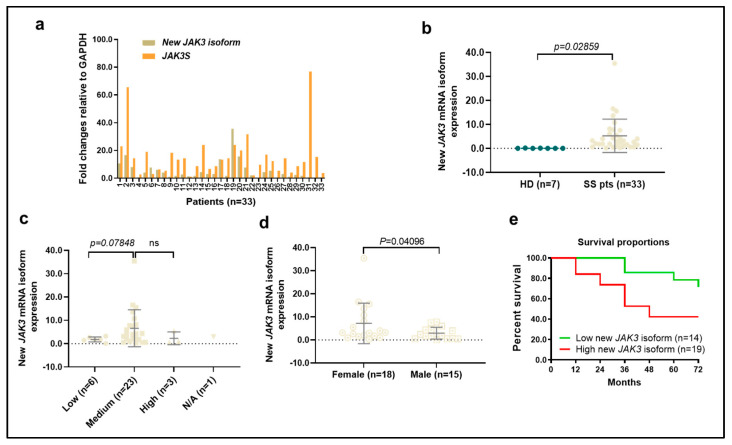
Expression of new *JAK3-INSL3* fusion transcript in different groups of SS patients. (**a**) Expression of both *JAK3-INSL3* fusion transcript and JAK3S mRNA were semi-quantified by qPCR in 33 SS patients; (**b**) Expression of *JAK3-INSL3* fusion transcript was different between SS patients (n = 33) and healthy donors (HD, n = 7) (unpaired *t*-test, *p* = 0.0286); (**c**) Expression of *JAK3-INSL3* fusion transcript was different among patients with different SS cell counts; (**d**) Expression of *JAK3-INSL3* fusion transcript was different between female (n = 18) and male (n = 15) patients (unpaired *t*-test, *p* = 0.041); (**e**) The expression levels of *JAK3-INSL3* fusion transcript were correlated with the overall survival of SS patients; Gehan-Breslow-Wilcoxon test, *p* = 0.0359.

We next assessed the expression levels of the *JAK3* and *JAK3-INSL3* fusion transcript by qPCR in all 33 SS patients. As a result, both the JAK3 and *JAK3-INSL3* fusion transcript were detected in all 33 patients, and the expression levels were very heterogeneous (Figure 2a). The range of the *JAK3-INSL3* fusion transcript was a 0.14~35.4 fold-change among all patients with an average of a 5.25 ± 6.85 fold-change. Patients had significantly higher expression of the *JAK3-INSL3* fusion transcript than healthy donors (Figure 2b). Five of seven healthy donors were negative for the *JAK3-INSL3* fusion transcript, and two healthy donors had nearly zero expression (0.08 ± 0.06 fold-change). Patients with high SS cells counts had a higher expression than patients with low SS counts, but patients with medium SS cell counts had the highest levels in comparison with patients with low or remarkably high SS cell counts (Figure 2c). Interestingly, female patients had higher expression levels than male patients (Figure 2d). Five patients who had a >10 fold-change expression of the *JAK3-INSL3* fusion transcript were all female. We also noticed that, on average, patients with large cell transformation (LCT) showed slightly higher expression (n = 13, 6.51 ± 5.66) than patients without LCT (n = 20, 4.42 ± 8.78, *p* = 0.191). Patients with SS that progressed from MF had slightly higher expressions (n = 8, 6.12 ± 6.10) than patients with de novo SS (n = 25, 4.96 ± 7.05, *p* = 0.338). 

Of interest, there was a correlation between the expression levels of *JAK3-INSL3* fusion transcript and patient overall survival. The 5-year survival of patients with a >-fold-change of *JAK3-INSL3* fusion transcript (n = 19) was 42.1% in comparison with 78.6% in patients with a ≤2 fold-change of expression (n = 14) (Gehan-Breslow-Wilcoxon test, *p* = 0.0359) (Figure 2e). These results support that our newly identified *JAK3-INSL3* fusion transcript has an oncogenic role in MF/SS CTCL. 

### 3.3. Knockdown of JAK3-INSL3 Fusion Transcript Inhibited Cell Proliferation and Colony Formation in Both MJ and HH Cells

**Figure 3:**
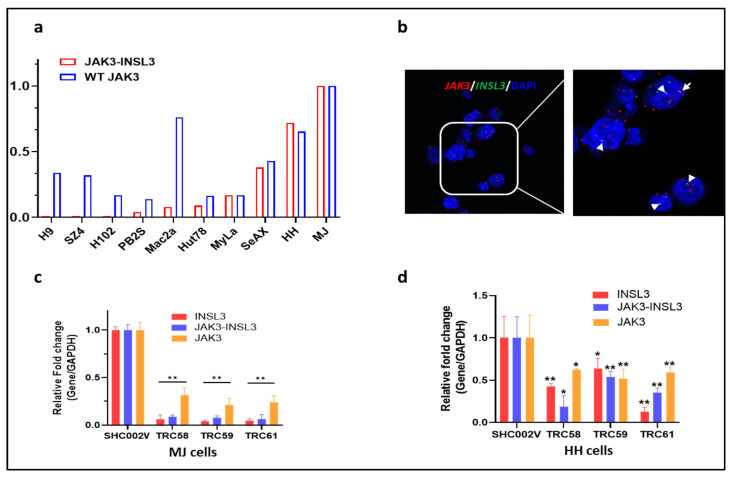
Expression of new *JAK3-INSL3* fusion transcript in CTCL cell lines and knockdown of *JAK3-INSL3* fusion transcript by *INSL3* shRNAs. (**a**) Expression of both *JAK3-INSL3* fusion transcript and JAK3S mRNA were semi-quantified by qPCR in 10 CTCL cell lines; (**b**) ViewRNA ISH Cell Assay was used to visualize the new *JAK3-INSL3* fusion transcript inside MJ cells, with the JAK3-specific probe in red and the INSL3-specific probe in green. Each set of red and green “dots” side by side corresponds to a single copy of the *JAK3-INSL3* fusion transcript (indicated by the arrows). Nuclei (blue) were stained with DAPI; (**c**) Expression of *INSL3*, *JAK3-INSL3*, and *JAK3* mRNAs were reduced in MJ cells after transduction with *INSL3* shRNAs (TRC58, TRC59, or TRC61) in comparison with cells transduced with non-target shRNA (SHC002V); (**d**) Expression of *INSL3*, *JAK3-INSL3*, and *JAK3* mRNAs were reduced in HH cells after transduction with *INSL3* shRNAs in comparison with cells transduced with control shRNA. Results represent the mean ± SD of triplicate determination: * *p* < 0.05; ** *p* < 0.01.

To further study the oncogenic roles of the new *JAK3-INSL3* fusion transcript in CTCL, we examined the expression of the *JAK3-INSL3* fusion transcript as well as wild type JAK3 in 10 CTCL cell lines. The expression of the *JAK3-INSL3* fusion transcript was heterogenous in different CTCL cell lines, and MJ and HH cells had the highest expressions among 10 cell lines (Figure 3a). We thus selected MJ and HH cells for our next experiments. Using the *JAK3*-specific probe (type 1 dye, red) and the *INSL3*-specific probe (type 4 dye, green), we were able to visualize the new *JAK3-INSL3* fusion transcript in situ in MJ cells with ViewRNA ISH Cell Assay. From Figure 3b, you can see several copies of *JAK3-INSL3* fusion transcript in MJ cells. Each set of red and green “dots” side by side corresponds to a single copy of the *JAK3-INSL3* fusion transcript. Nuclei were stained blue with DAPI. 

We then conducted gene-silencing experiments using RNA interference. It is known that INSL3 is exclusively expressed in testes and barely expressed in other tissues, including hematopoietic cells [29]. When we failed to design effective short hairpin RNAs (shRNAs) to target the fusion site of the new *JAK3-INSL3* fusion transcript, we selected/tested five predesigned shRNAs targeting exon 1 or/and exon 3 of *INSL3* instead (Table S4). The effectiveness of the gene knockdown was confirmed using qPCR for expression of *INSL3* and *JAK3-INSL3*. The expression of *JAK3* was also assessed. As a result, the *INSL3* shRNAs tested were effective in the downregulation of expression of *INSL3* as well as *JAK3-INSL3*. As shown in Figure 3c,d, both the MJ and HH cells transduced with *INSL3* shRNAs (TRC58, TRC59, and TRC61) had big decreases in the expression of *INSL3* as well as *JAK3-INSL3* in comparison with the non-target shRNA control (SHC002V). Of note, most of *INSL3* shRNAs showed fewer effects on *JAK3* expression in comparison with its effect on *INSL3* and *JAK3-INSL3* expression.

**Figure 4:**
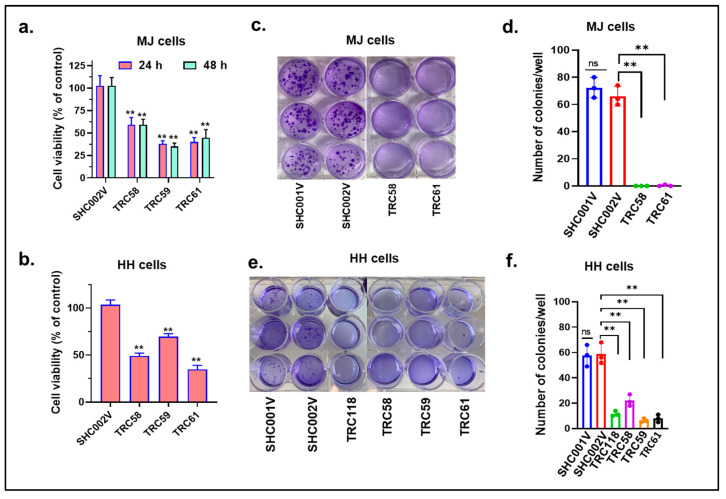
Knockdown of new *JAK3-INSL3* fusion transcript inhibited cell proliferation and colony formation in MJ and HH cells. (**a**) CellTiter-GloTM Luminescent Cell Viability Assay was used to assess cell viability at 24 h and 48 h for MJ cells transduced with TRC58, TRC59, TRC61 *INSL3* shRNAs or non-target shRNA (SHC002V). The cell viability was reduced in MJ cells transduced with *INSL3* shRNAs in comparison with cells transduced with control shRNA; (**b**) The cell viability at 24 h was reduced in HH cells transduced with TRC58, TRC59, TRC61 *INSL3* shRNAs in comparison with cells transduced with control shRNA; (**c**) Soft agar colony formation assay was performed using MJ cells transduced with TRC58, TRC61 *INSL3* shRNAs or vector only SHC001V or SHC002V control shRNAs; (**d**) The numbers of colonies were much fewer in MJ cells transduced with *INSL3* shRNAs in comparison with cells transduced with control shRNAs; data are representative of three biological replicates; (**e**) Soft agar colony formation assay was performed with HH cells transduced with TRC58, TRC59, TRC61, and TRC118 *INSL3* shRNAs or control shRNAs; (**f**) The numbers of colonies were much fewer in HH cells transduced with INSL3. Results represent the mean ± SD of triplicate determination: ns, not significant; ** *p* < 0.01.

We then assessed the effects of gene knockdown on the cell viability, cell proliferation, and colony formation in these cells. First, there were big decreases in cell numbers after transduction for 1 week in both the MJ and HH cells transduced with *INSL3* shRNAs than in cells transduced with the control shRNAs. The cells transduced with TRC58- and TRC61-shRNAs had even lower numbers than other *INSL3* shRNAs. We next assessed the cell proliferation of different cells with a CellTiter-Glo cell viability assay. As shown at Figure 4a,b, the proliferation of cells transduced with *INSL3* shRNAs was inhibited in both the MJ and HH cells at 24 h and/or 48 h in comparison with cells transduced with control shRNAs. We finally assessed the capability of colony forming between these cells over a 2-week period. Figure 4c,d shows that the colony numbers in MJ cells transduced with TRC58- and TRC61-*INSL3* shRNAs were dramatically decreased in comparison with cells transduced with control shRNAs (SHC001V and SHC002V). Similar inhibitory patterns were also observed in *INSL3* shRNA transduced HH cells (Figure 4e,f). We also further conducted gene knockout experiments using CRISP/CAS9 editing in MJ cells. Our findings were consistent with our results from the gene knockdown above. The decreased cell proliferation and colony formation were observed in MJ cells with gene knockout (Supplementary materials: Figure S1, Table S5). Taken together, our results suggest that the knockdown or knockout of the new *JAK3-INSL3* fusion transcript in both the MJ and HH cells inhibits cell proliferation and colony formation and support its oncogenic role in MF/SS CTCL.

### 3.4. Knockdown of JAK3-INSL3 Fusion Transcript in MJ and HH Cells Downregulated the JAK3/STATs/NF-κB Signaling Pathways 

**Figure 5:**
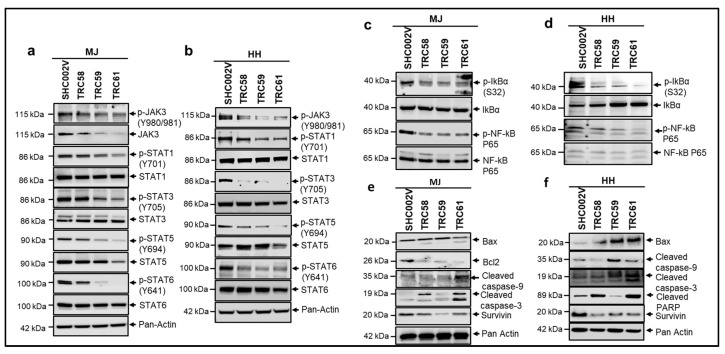
Knockdown of new *JAK3-INSL3* fusion transcript downregulated JAK3/STATS/NF-κB signaling pathways and induced apoptosis in MJ and HH cells. Whole cell protein was extracted from cells transduced with *INSL3* shRNAs (TRC58, TRC59, and TRC61) as well as non-target shRNA (SHC002V) for Western blot analysis using antibodies specific for JAK3/STATS/NF-κB molecules indicated. (**a**) Western blot showed that JAK3, p-JAK3, p-STAT1, p-STAT3, STAT5, p-STAT5, and p-STAT6 were decreased in TRC58-, TRC59-, and TRC61-transduced MJ cells versus SHC002V transduced cells; (**b**) Western blot showed that p-JAK3, p-STAT1, p-STAT3, p-STAT5, and p-STAT6 were decreased in TRC58-, TRC59-, and TRC61-transduced HH cells versus SHC002V-transduced cells; (**c**) Western blot showed that p-IkBa and p-NF-kB P65 were decreased in TRC58, TRC59, and TRC61-transduced MJ cells versus SHC002V-transduced cells; (**d**) Western blot showed that p-IkBa and p-NF-kB P65 were decreased in TRC58-, TRC59-, and TRC61-transduced HH cells versus SHC002V-transduced cells; (**e**) Western blot showed that anti-apoptosis proteins, survivin and Bcl2, were decreased, and pro-apoptosis molecule, Bax, and apoptotic-cleaved proteins were increased in TRC58-, TRC59-, and TRC61-transduced MJ cells versus SHC002-transduced cells; (**f**) Western blot showed that survivin was decreased, and Bax and apoptotic-cleaved proteins were increased in TRC58-, TRC59-, and TRC61-transduced HH cells versus SHC002V-transduced cells.

The constitutive activation of the JAK3/STATs/NF-κB pathway contributes to the pathogenesis of CTCL [30,31,32,33]. We thus examined the effects of knockdown of *JAK3-INSL3* fusion transcript on JAK3/STATs/NF-κB signaling pathways in both the MJ and HH cells. Levels of involved proteins were assessed by Western blots. MJ cells stably transduced with *INSL3* shRNAs (TRC58, TRC59, and TRC61) showed lower levels of proteins of JAK3, STAT3, and STAT5 than cells transduced with SHC002V shRNA. Of note, many phosphorylated proteins (p-STAT1, p-STAT3, p-STAT5, and p-STAT6) were reduced much more than total proteins (Figure 5a). HH cells also showed similar patterns. HH cells transduced with *INSL3* shRNAs had lower p-JAK3, p-STAT1, p-STAT3, p-STAT5, and p-STAT6 than cells transduced with SHC002V (Figure 5b). Western blot analysis also showed decreased levels of p-IκBα and p-NF-κB p65 in both the MJ and HH cell lines, as shown in Figure 5c,d.

The constitutive activation of the NF-κB pathway not only contributes to increased cell proliferation, but also to the apoptosis resistance of lymphoma cells in CTCL [32]. We thus also assessed proteins involved in cell apoptosis by using Western blots. As shown in Figure 5e,f, the level of Bax, a pro-apoptotic protein, was higher or induced in *INSL3* shRNA-transduced MJ and HH cells than in the control-transduced cells. Meanwhile, the levels of Bcl-2 and/or survivin, anti-apoptotic proteins, were decreased in *INSL3* shRNA-transduced cells. In addition, cleaved caspase-9, cleaved caspase-3, and/or cleaved PARP, the hallmark apoptosis proteins, were increased in the *INSL3* shRNA-transduced MJ and HH cells. These results suggest that the knockdown of the *JAK3-INSL3* fusion transcript downregulates the JAK3/STATs/NF-κB signaling pathway and may promote cell apoptosis in MJ and HH cells.

**Figure 6:**
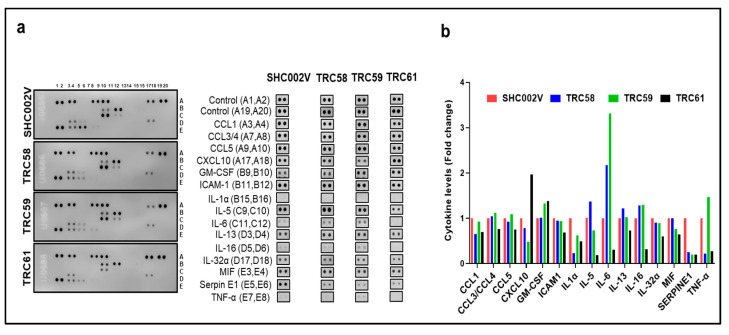
Knockdown of new *JAK3-INSL3* fusion transcript inhibited and moderated cytokines/chemokines in MJ cells. (**a**) Cell lysates from TRC58, TRC59, TRC61 or SHC002V transduced cells were used to detect cytokines/chemokines using the Human Cytokine Array. The duplicate spots correspond to the cytokines/chemokines which were altered were shown; (**b**) Densitometric analysis of the dot blot duplicates from panels; fold changes were plots for 15 cytokines/chemokines in 4 groups.

It is known that the constitutive activation of the JAK3/STATs/NF-κB pathway is related to abnormal cytokines/chemokines or Th1/Th2 shift in CTCL [33]. Thus, we examined/compared the cytokine/chemokine profiles by using a Human Cytokine Array in different MJ cells (Figure 6a). As shown in Figure 6b, multiple cytokines/chemokines were reduced in the MJ cells transduced with *INSL3* shRNAs compared to the cells transduced with control shRNA, such as CCL1, ICAM1, IL1α, IL-32α, and SERPIN1. But, IL5, IL6, IL-13, IL16, MIPα, MIF, and CCL5 were mainly decreased in TRC061-transduced cells, while TNFα was significantly decreased in TRC058- and TRC061-transduced cells. These results suggest that the knockdown of the *JAK3-INSL3* fusion transcript may inhibit and moderate the abnormal cytokines/chemokines in MJ cells.

### 3.5. Knockdown of JAK3-INSL3 Fusion Transcript Inhibited Tumor Formation and Growth in NSG Mice In Vivo

Our in vitro results above show that the *JAK3-INSL3* fusion transcript was efficiently knocked down by *INSL3* shRNAs in both the MJ and HH cell lines. Also, cells with the *JAK3-INSL3* fusion transcript stably knocked down had reduced cell proliferation and decreased colony formation. Next, we evaluated the effects of the knockdown of the *JAK3-INSL3* fusion transcript in MJ cells on tumor formation and growth in NSG mice in vivo. MJ cells with *JAK3-INSL3* stable knockdown (TRC058 cells or TRC061 cells) or control cells (SHC002V cells) were subcutaneously injected into 6–8-week-old NSG mice (three groups with three mice in each group). Tumor formation and growth were monitored twice a week or as needed.

**Figure 7:**
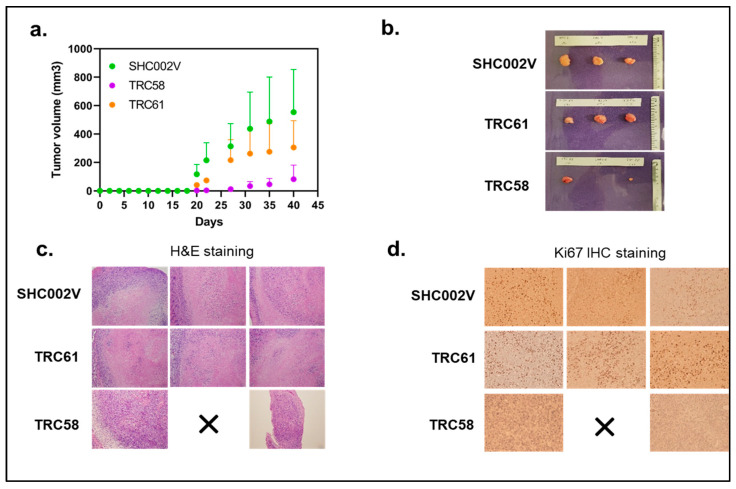
Knockdown of new *JAK3-INSL3* fusion transcript in MJ cells inhibited xenograft tumor formation and growth in NSG mice. (**a**) MJ cells transduced with *INSL3* shRNA (TRC58 and TRC61) and non-target shRNA-SHC002V were subcutaneously injected into NSG mice (three mice/group). Tumor formation and growth were monitored and measured twice a week or as needed, and tumor volumes (mm3) were calculated until sacrifice; (**b**) Upon mouse sacrifice, tumors were removed, measured, and weighted; tumors were then processed and embedded in paraffin blocks; (**c**) Sections were cut and stained with hematoxylin and eosin (H&E) for histological evaluation; (**d**) Ki-67 protein expression was analyzed using immunohistochemistry.

Small tumors were visible in eight out of nine mice at Day 20 after cell injection, and only one mouse injected with TRC058 cells (TRC058 mice) had no tumors visible. The average volumes of the tumor masses on Day 20 were 2.8 ± 2.8 mm^3^ (n = 2) in TRC058 mice, 41.0 ± 14.2 mm^3^ (n = 3) in TRC061 mice, and 117.2 ± 55.8 mm3 (n = 3) in SHC002V mice. Clearly, tumor masses in TRC058 mice and TRC061 mice were smaller than those in SHC002V mice. TRC058 mice had the smallest tumors (*p* = 0.022). From Figure 7a, you can see the growth of tumors in the three groups of mice were different. On Day 40, the average volume of the tumor masses was 82.2 ± 81.4 mm^3^ (n = 2) in the TRC058 mice, still the smallest (*p* = 0.031) among three groups. Also, the same TRC058 mouse had no tumor mass formation from the beginning to the end. The average tumor volume in TRC061 mice (305.1 ± 154.6 mm3, n = 3) was still smaller than that in SHC002V mice (554.2 ± 245.3 mm^3^, n = 3, *p* = 0.146). The mice were sacrificed on Day 40, and the tumors were removed for H&E and further analysis (Figure 7b,c). Ki-67 positive cells from immunohistochemical staining in tumor lesions from TRC058 mice were significantly reduced compared to those from SHC002V mice, as shown in Figure 7d. The difference in the Ki-67 positive cells in the tumor lesions between the TRC061 mice and SHC002V mice was small. Our results here suggest the knockdown of the *JAK3-INSL3* fusion transcript in MJ cells inhibits tumor formation and growth in NSG mice in vivo.

## 4. Discussion

In this study, we report a newly identified *JAK3-INSL3* fusion transcript in MF/SS CTCL. SS patients with a high-level expression of the *JAK3-INSL3* fusion transcript had poorer survival than SS patients with a low-level expression. Knockdown or knockout of the *JAK3-INSL3* fusion transcript in MJ and HH cells led to a reduced cell proliferation, decreased colony formation, and delayed/reduced tumor formation in NSG xenograft mice. These results suggest that the newly identified *JAK3-INSL3* fusion transcript is oncogenic, and it may contribute to constitutive JAK3 activation and lymphomagenesis in MF/SS CTCL.

Fusion transcripts of adjacent genes were thought to originate only from chromosomal rearrangements, but the discovery of read-through transcripts has updated our perception in the past years. Advances in sequencing technologies have allowed for better detection of fusion transcripts, and many fusion transcripts that contain some exons of both the upstream and downstream genes originate from intergenic cis-splicing or trans-splicing process [12]. Although not all fusion transcripts are inherently oncogenic and some have been observed in healthy cells or tissues, studies report that fusion events drive 16.5% of human cancers and function [34]. Also, recurrent fusions involving kinases have been often discovered [12]. Multiple fusion transcripts have recently been detected in CTCL by different groups [6,7,9]. Using RNAseq analysis, our group identified a set of fusion transcripts, such as *C15orf57-CBX3*, *MDD22-SURF6*, and *CD28-CTLA4*, in sorted malignant T-cells from SS patients [5]. The reason we selected the *JAK3-INSL3* fusion transcript for validation is because of its high incidences and constitutive JAK3 activation implicated in the pathogenesis of CTCL.

Constitutive JAK3 activation has been found in different lymphoid malignancies, including mantle-cell lymphoma [35], Burkitt lymphoma [36], anaplastic large-cell lymphoma [37], HTLV-1-induced adult T-cell lymphoma/leukemia [38], and CTCL [24,39], but the mechanisms of constitutive JAK3 activation are still largely unknown. The activating JAK3A572V mutation has been reported to elucidate the effects of constitutive JAK3 in CTCL, but it only accounts for 3% of patients [25]. Recent studies suggest that fusion transcripts may participate in constitutive JAK3 activation and promote its downstream signaling pathways of cancer cells [40]. Our results in this study show that the knockdown of the *JAK3-INSL3* fusion transcript in CTCL cell lines decreased phosphorylated JAK3 and its downstream STAT/NF-κb signaling pathways. Furthermore, cells, after the knockdown of *JAK3-INSL3* had abrogated cell viability, decreased cell proliferation and colony-forming ability, as well as reduced tumor growth in vivo. Our findings support our hypothesis that the newly identified *JAK3-INSL3* fusion transcript contributes to constitutive JAK3 activation, and this could be an additional mechanism of constitutive JAK3 activation in MF/SS CTCL. Although more studies need be performed to uncover the mechanistic details, this potential mechanism expands our understanding of constitutive JAK3 activation in MF/SS CTCL and fills the knowledge gap.

Clinical findings in this study are very interesting. The levels of newly identified *JAK3-INSL3* fusion transcript expression were inversely correlated with the patient’s survival. Of note, the highest levels of *JAK3-INSL3* fusion transcript expression were seen in female patients. In addition, patients with large cell transformation (LCT) and patients with SS progressed from MF tended to have higher expression of *JAK3-INSL3* fusion transcript than those without LCT or patients with de novo SS. Therefore, our newly identified *JAK3-INSL3* fusion transcript has great clinical implications and may serve as a biomarker for patient grouping, molecular diagnosis, and prognosis in the future.

Aberrant JAK3 activation and its downstream signaling pathways are implicated in the pathogenesis of CTCL, and inhibition of JAK3 has been a therapeutic strategy for CTCL with increasing attentions. Many studies have shown that JAK3 inhibitors had good effects in CTCL cell lines [15,41,42]. For example, Tofacitinib, a JAK3 selective inhibitor, showed inhibitory effects on intracellular growth factor and cytokine-mediated signals. The clinical trials of JAK3 inhibitors are also on the way for CTCL patients. We believe that the more we uncover mechanisms of constitutive JAK3 activation, the more therapies can be developed. Based on our findings in this study, the *JAK3-INSL3* fusion transcript may also serve as a potential therapeutic target in MF/SS CTCL in the future.

There are some weaknesses and limitations in this study. The sample size of our mouse experiments is small and included only three mice in each group. In fact, we conducted the second experiment with an increased sample size (seven mice for each group) after the first mouse experiment. Unfortunately, the pandemic occurred, and the lab was shut down at that time. Experiments for cell transduction in vitro and in vivo mouse work were largely affected and failed.

In fact, our findings in this study come mostly from SS patients and MF/SS-derived CTCL cell lines. Whether a new *JAK3-INSL3* fusion transcript may also be an oncogenic event in non-MF/SS CTCLs will be further studied. In addition, as mentioned above, constitutive JAK3 activation is not only found in CTCL patients but also in some other lymphoid malignancies. Future studies to access the expression of *JAK3-INSL3* fusion transcripts and its oncogenic roles in these lymphoid malignancies will be interesting. Also, multiple fusion transcripts have been detected by our RNAseq analysis in SS patients, although incidences of most of them are less frequent. Further studies to assess and validate them will help us uncover their roles in CTCL and may also help CTCL transcriptional subtyping or even future personalized therapy.

## Data Availability

All data generated and analyzed during this study are included in this article.

## Supplementary Materials

The following supporting information can be downloaded at: https://www.mdpi.com/article/10.3390/cells12192381/s1, Figure S1: Knockout of *JAK3-INSL3* fusion transcript inhibited cell proliferation and colony-formation in MJ cells; Table S1: Patient demographics; Table S2: Two types of *JAK3-INSL3* fusion transcripts were detected by RNAseq in SS patients; Table S3: PCR primers; Table S4: *INSL3* shRNAs; Table S5: *INSL3* sgRNAs; Table S6: Antibodies used for Western blotting.
