# Supplementary material for: New JAK3-INSL3 Fusion Transcript—An Oncogenic Event in Cutaneous T-Cell Lymphoma
Source: Cells. 2023 Sep 29;12(19):2381. doi: 10.3390/cells12192381 (PMC10572011; doi:10.3390/cells12192381)
Supplement: Supplementary file 1 [file cells-12-02381-s001.zip › Supplimentory figure f.pptx]

## Slide 1
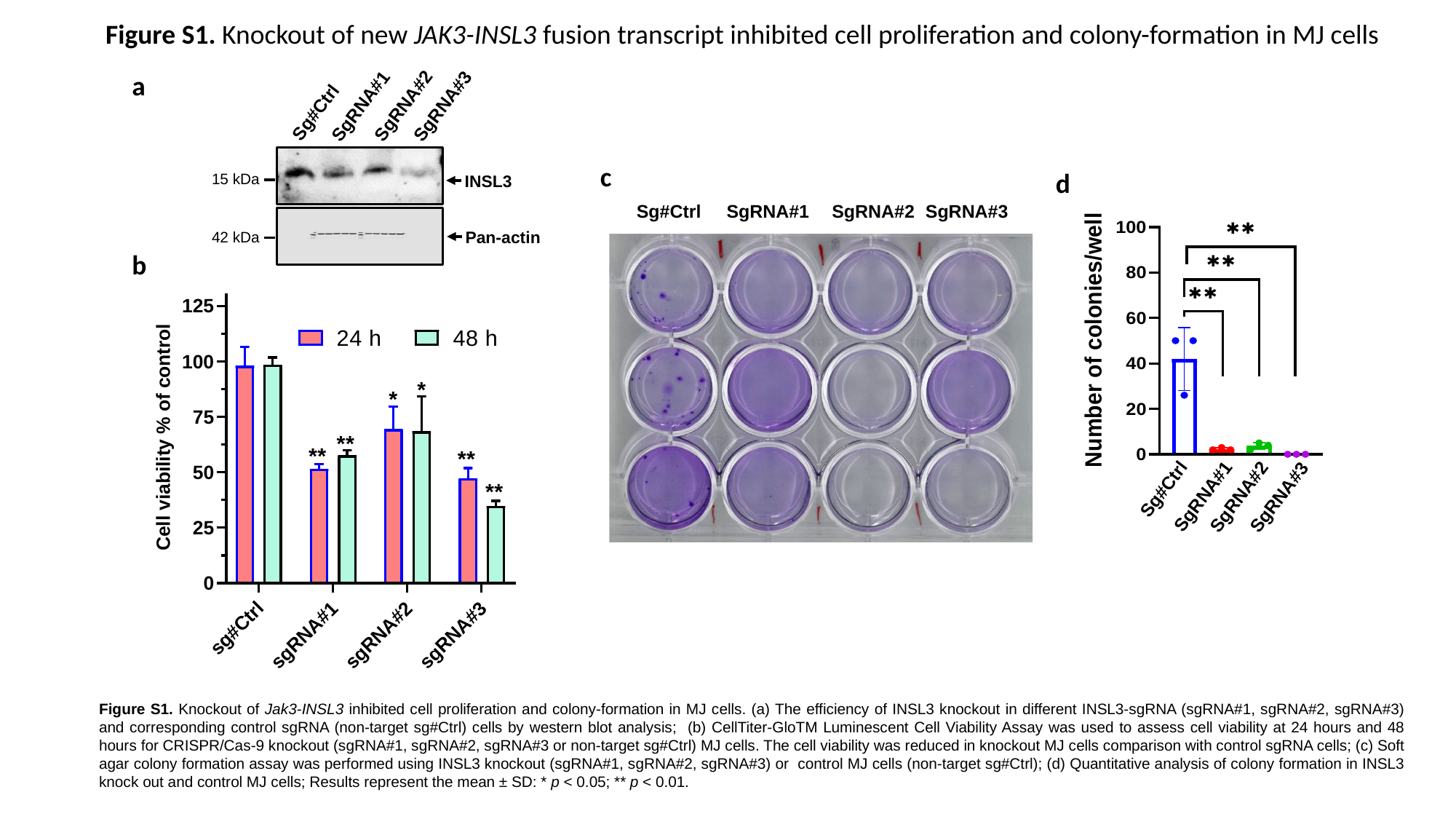

Figure S1. Knockout of new JAK3-INSL3 fusion transcript inhibited cell proliferation and colony-formation in MJ cells
SgRNA#2
SgRNA#1
SgRNA#3
Sg#Ctrl
INSL3
Pan-actin
 15 kDa
 42 kDa
a
c
d
SgRNA#2
SgRNA#3
SgRNA#1
Sg#Ctrl
Sg#Ctrl
SgRNA#1
SgRNA#2
SgRNA#3
b
Figure S1. Knockout of Jak3-INSL3 inhibited cell proliferation and colony-formation in MJ cells. (a) The efficiency of INSL3 knockout in different INSL3-sgRNA (sgRNA#1, sgRNA#2, sgRNA#3) and corresponding control sgRNA (non-target sg#Ctrl) cells by western blot analysis; (b) CellTiter-GloTM Luminescent Cell Viability Assay was used to assess cell viability at 24 hours and 48 hours for CRISPR/Cas-9 knockout (sgRNA#1, sgRNA#2, sgRNA#3 or non-target sg#Ctrl) MJ cells. The cell viability was reduced in knockout MJ cells comparison with control sgRNA cells; (c) Soft agar colony formation assay was performed using INSL3 knockout (sgRNA#1, sgRNA#2, sgRNA#3) or control MJ cells (non-target sg#Ctrl); (d) Quantitative analysis of colony formation in INSL3 knock out and control MJ cells; Results represent the mean ± SD: * p < 0.05; ** p < 0.01.
